# Patterning, Reading, and Executive Functions

**DOI:** 10.3389/fpsyg.2018.01802

**Published:** 2018-09-25

**Authors:** Allison M. Bock, Kelly B. Cartwright, Patrick E. McKnight, Allyson B. Patterson, Amber G. Shriver, Britney M. Leaf, Mandana K. Mohtasham, Katherine C. Vennergrund, Robert Pasnak

**Affiliations:** ^1^Department of Psychology, George Mason University, Fairfax, VA, United States; ^2^Department of Psychology, Neuroscience and Teacher Preparation, Christopher Newport University, Newport News, VA, United States

**Keywords:** reading, patterning, executive function, cognitive flexibility, working memory

## Abstract

Detecting a pattern within a sequence of ordered units, defined as patterning, is a cognitive ability that is important in learning mathematics and influential in learning to read. The present study was designed to examine relations between first-grade children’s executive functions, patterning, and reading abilities, and to examine whether these relations differ by the type of pattern. The results showed that working memory correlated with reading fluency, and comprehension measures. Inhibition correlated only with the latter. Cognitive flexibility was correlated with patterning performance and with performance on object size patterns, whereas working memory was correlated with performance on symmetrical patterns and growing number patterns. These results suggest that the cognition required for completing patterns differs depending on the pattern type. Teachers may find it beneficial to place emphasis on the switching and working memory components of completing patterning tasks, depending on the type of patterns used in instruction.

## Introduction

What form of instructionintended to improve children’s thinking is most widely employed in elementary schools in English-speaking countries? What such instruction have most English-speaking psychologists younger than 50 received but never heard of since? The answer is “patterning.” As commonly employed, patterning instruction consists of teaching young children that series of alternating items follow a consistent rule that makes all of the series alike even though they consist on different elements. Examples are red blue red blue red blue or circle square circle square circle square or large small large small large small. The series can become more complicated, e.g., double alternations or alternations of three elements. The theory and research behind children’s understanding of such patterns has been almost exclusively the province of educators, although there have recently been studies of children’s learning of alternating 4-item temporal patterns (Lange-Kuettner et al., 2012; Hentschel et al., 2016). For more than 50 years patterning has been taught in the vast majority of preschools and kindergartens in most English-speaking countries. Improving children’s understanding of patterns is thought to improve cognition, in contrast to other preschool activities – learning numbers, counting, writing letters – that are aimed more directly at academic achievement.

Detecting a pattern among a set of units is an underlying ability that supports children’s development of mathematics concepts and (perhaps) reading (Papic and Mulligan, 2005; Burgoyne et al., 2017; Schmerold et al., 2017). In the United States, the National Council of Teachers of Mathematics (2006) and the joint position statement of the National Association for the Education of Young Children/National Council of Teachers of Mathematics Education (2002/2010) recommended helping children to understand patterns, to help ensure that children are prepared for mathematical reasoning. An opposing view, recommending reduction of emphasis on patterning, has been presented by the National Mathematics Advisory Panel (2008). However, patterning with alternating numbers and blocks has been commonly taught within preschool and elementary school mathematics curricula for many years (Clements and Sarama, 2007a). The brief review by Burgoyne et al. (2017) and a more extensive review by Pasnak (2017) summarize evidence from several studies indicating that patterning is related to mathematics, and that children’s understanding of repeating patterns (simple alternating pattern of sizes, colors, or shapes) in kindergarten is predictive of later mathematics achievement in fifth grade (Rittle-Johnson et al., 2017). There is also an influential study (Lee et al., 2011) of relations between older (10- and 11-year-olds) children’s understanding of very complex number patterns and their understanding of algebra.

However, a more general understanding of complex patterns by older children apparently influences a broader range of academic skills than mathematics (Hendricks et al., 2006; Kidd et al., 2014; Pasnak et al., 2015). These studies, which employed different standardized tests, indicated that patterning may also be a precursor for early reading skills. In these studies, first-grade children were taught how to detect increasing, decreasing, or symmetrical patterns with letters, numbers, clocks, or objects, or with objects that rotate. Patterns became more complicated and varied over time to ensure strengthening of children’s general patterning understanding. Children learned to detect which item came next in each type of pattern. Following the interventions, children showed significant gains in patterning, mathematics, and reading achievement assessed by measures of word reading, reading fluency, and reading comprehension. These findings suggest that patterning is a cognitive ability that influences a broad range of academic skills.

Examining the cognitive abilities needed to understand patterns can help inform the most effective ways to teach children about patterns as well as suggest interventions that can be implemented when children struggle. Therefore, one goal of the current study was to develop more evidence of what abilities may underlie patterning. We first examine previous research on this topic.

### Patterning and Other Cognitive Abilities

Comprehending patterns involves the ability to detect the regularity in a sequence. Patterning is theorized to require generalization and abstract reasoning about the stimuli in the pattern (Clements and Sarama, 2007b). Preschoolers can solve simple repeating patterns (those with alternating elements, e.g., red blue red blue red blue) by one-to-one appearance matching or by relational thinking. The latter requires the ability to make comparisons across two things (Collins and Laski, 2015; Fyfe et al., 2015), and also functional thinking – the ability to understand how two quantities vary (Blanton and Kaput, 2011).

Recent work examining the cognition involved in patterning has focused on the role that executive functions (EFs) may play in patterning ability. EF are cognitive abilities that underlie goal-directed behavior. These include inhibition – resisting both irrelevant information and impulsive behavior; working memory, – holding and manipulating information in one’s mind; and cognitive flexibility, – shifting one’s thinking based on rules or demands (Miyake et al., 2000). EF first develop during the preschool years, i.e., between 3 and 5 years of age (Carlson et al., 2016). Bull and Lee (2014) suggested that these functions may emerge as a unitary construct in preschool. Throughout early and middle childhood, children’s EF continue to develop and become more distinct from one another (Best and Miller, 2010). Most researchers agree that children’s EF involve at least three primary components: working memory, inhibition, and cognitive flexibility (Carlson et al., 2016).

Research examining the EF involved in preschoolers’ understanding of repeating patterns has shown that cognitive flexibility alone (Bennett and Muller, 2010), or both cognitive flexibility and working memory (Miller et al., 2015; Harvey and Miller, 2017), or inhibition and working memory (Collins and Laski, 2015) are related to understanding patterns. For older children, cognitive flexibility correlated with first-grade children’s understanding of computer generated matrix patterns and with a global measure of complex letter, number, rotation, and shape patterns (Bock et al., 2015); Schmerold et al. (2017) showed that both cognitive flexibility and working memory correlated with the latter patterning measure.

Brydges et al. (2012) found that, for 7- to 10-year-old children, the ability to detect patterns within items in a matrix (e.g., as assessed by Raven’s Progressive Matrices test, RPM), was best predicted by an overall composite of EF (and is related to all EF variables, with working memory being the strongest factor. These matrix-patterning assessments often involve patterns with different types of shapes; however, patterns may vary drastically within the assessment, as items within a pattern may vary in coloring, orientation, and size. For still older children (10-year-olds) working memory predicted patterning performance on complex number patterns (Lee et al., 2011).

Overall, these findings suggest that children may use employ working memory or cognitive flexibility, or both of them, or all EF skills when detecting pattern sequences. However, the lack of consistency between experimental outcomes, especially in the elementary school years, may be dependent on the type of pattern that is used to assess pattern understanding, which may involve such varied stimuli as numbers, letters, matrices, or shapes. One goal of the current study is to examine whether different types of patterns are differentially related to EF.

### Types of Patterns

Patterns may vary in a number of ways, including the type of regularity and the content. For example, patterns may have units that repeat, are symmetrically ordered, or grow (ascend or descend). The content may include different colors, numbers, sizes, and shapes. During the preschool years, children develop understanding of repeating patterns, which involves the ability to replicate and extend these patterns (Miller et al., 2015). Patterns that are taught to preschoolers usually are repetitions of different shapes, colors, or sizes. In the early elementary school years, children begin to understand more challenging patterns, including growing patterns, which may include increasing or decreasing numbers, or letters which come progressively later or earlier in the alphabet. Research with elementary school children has focused on either a variety of patterns, including letters, numbers, and object size (Kidd et al., 2014; Bock et al., 2015), or focused solely on numbers (Lee et al., 2011). However, growing numerical patterns may differ uniquely from other types of patterns, and may be supported by unique cognitive skills (Liljedahl, 2004). One aspect of some previous studies of the effects of patterning instruction has been the use of a variety of patterns so that children can strengthen their patterning ability to generalize to a variety of situations (Hendricks et al., 2006; Kidd et al., 2013, 2014; Pasnak et al., 2015). Gadzichowski’s (2012a,b) pioneering studies indicated that there were no significant differences in first graders’ ability to learn patterns composed of letters, numbers, colors, objects, or shapes, but that there were significant differences between the ease with which they learned different types of patterns, with repeating patterns being easiest. Gadzichowski et al. (2014) later found that orientations of patterns made a difference, with letter patterns being easier when in horizontal orientations (rows) and number patterns being easier when presented in vertical orientations (columns). The effects were strongest for the most difficult patterns. However, no researchers have examined whether different types of patterns differentially affect learning or require separate cognitive abilities.

### Patterning and Reading

The case for a relationship between patterning and mathematics has become increasingly clear, with the longitudinal study of repeating pattern knowledge by Rittle-Johnson et al. (2017), the correlational study of Lee et al. (2011) and the studies of Kidd et al. (2013, 2014) and Pasnak et al. (2015) all converging to provide empirical evidence of the relationship. This was anticipated by Baroody (1993), Orton (1999), Warren et al. (2006), Clements and Sarama (2007a,b,c), Papic (2007), and many others.

The case for reading is, as Burgoyne et al. (2017) suggest, not quite so well established, but seems fairly strong. Hendricks et al. (2006) found a significant effect of instruction with a huge assortment of patterns on the written language scale of the Diagnostic Achievement Battery-2, and Kidd et al. (2014) found that an intervention also employing numerous types of patterns produced significant differences on the Gray Oral Reading Test-4 (GORT), the Test of Word Reading Ability (TOWRE), and the Test of Early Reading Ability-3 (TERA). Pasnak et al. (2015) replicated these results on the TERA and TOWRE but not the GORT. However, Kidd et al. (2013) reported that the same patterning intervention had no effect on three Woodcock–Johnson III reading scales. Hence, there is empirical evidence that reading and patterning are related. However, it is not consistent, and it is not clear what the functional relationship between reading and patterning might be. If there is indeed a relationship it may be purely correlational, as Pasnak et al. (2016) suggested and reflect some general form of intelligence. Alternatively, Pasnak et al. (2015) speculated that patterning may involve some aspect of the Grw broad spectrum component of the Cattel–Horn–Carroll theory of intelligence. This may be a default position. However, Manning et al. (1995) pointed out that there are sequences in sentence structure that may be helpful to beginning readers, and Waller’s (1977) review showed long ago that children’s proficiency in serial ordering was related to reading. Sarama and Clements’s (2004) Sarama and Clement’s (2004) call for more empirical investigation of potential relations between patterning and reading is still appropriate.

### Relations Between Patterning, Reading, and Executive Functions

Bock et al. (2015) found that first grader’s patterning scores on the complex patterning test used in these patterning studies correlated with TERA reading scores, and with both a card sort measure and a unique puzzle measure of cognitive flexibility. Schmerold et al. (2017) pursued the relationship between this patterning measure, EF, and both reading and mathematics. Patterning was related to cognitive flexibility, working memory, reading, and mathematics. Regression analyses and structural equation modeling showed that the effect of cognitive flexibility was entirely mediated by patterning, while working memory had independent effects as well as effects moderated by patterning.

Abreu et al. (2014) focused specifically on poor readers. Their subjects were 6- to 8-year-old Brazilian children, so they were roughly a year older than those studied by Bock et al. (2015) and Schmerold et al. (2017), and half lived below the Brazilian poverty line. A large variety of EF measures were administered. Principal components analysis identified four factors, inhibition, selective attention, interference suppression, and a factor combining working memory with cognitive flexibility. The latter factor was the only one which differentiated good readers from poor readers, as those were defined by their teachers. These researchers did not measure the children’s patterning ability, but the relation to the working memory/cognitive flexibility factor suggests the potential to a relation between reading and patterning for these 7-year-olds similar to those reported by Bock et al. (2015) and Schmerold et al. (2017) for 6-year-olds.

### The Current Study

The patterns used in the studies by Kidd et al. (2014), Bock et al. (2015), Pasnak et al. (2015), and Schmerold et al. (2017) were, by design, very complex and variable. There were five types of patterns which were presented either horizontally or vertically. Each of the five types was composed of five types of elements: letters, numbers, shapes, clock faces, and pictorial representations of objects. It is impossible to determine just what types of patterns were related to EF or reading. The current study is in part an attempt to identify more exactly what types of patterns are related to reading and to EF for first graders. Hence, we examined the relation between different types of patterns and EF, focusing on the three major EF components that have been most strongly linked to patterning. We hypothesized that general overall patterning across patterning types would be related to both working memory and cognitive flexibility. Additionally, performance on a patterning measure with fewer types of patterns than the set used by Kidd et al. (2014), Bock et al. (2015), Pasnak et al. (2015), and Schmerold et al. (2017) was examined for evidence of different relations between reading, EF, and types of patterns. It was hypothesized that:

(1) Patterns of numbers, letters, and shapes would be differentially related to reading and EF.
(2) Patterns of items that either ascended or descended in value, position in the alphabet, or size, or were symmetrical would be differentially related to reading and EF, and
(3) The above two factors would interact, so that the nature of the pattern (ascending, descending, or symmetrical) would interact with the elements of which it was composed to differentially relate to reading and EF.

## Materials and Methods

### Participants

The research was reviewed and approved in George Mason University’s Office of Sponsored Programs, Research Development, Integrity, and Assurance by the committee governing ethical conduct of university-sponsored research. Written informed consent was obtained from parents of 84 children from 11 first-grade classrooms in two public elementary schools in an urban Mid-Atlantic area. Children were excluded from the study if they had an Individualized Education Plan or were considered by their teachers to not be proficient in English. This sampling yielded an approximately equal number of boys (*N* = 40) and girls (*N* = 44) who were 6–7 years old. Birthdays were considered to be protected information, so exact ages could not be ascertained. Twenty-two percent were white, 28% were Latino/Hispanic, and 39% were African American.

### Measures

There was an assessment of reading ability, the GORT, an assessment of patterning ability, and three assessments of EF skills. These included the Day/Night inhibition test, the Multiple Classification Card Sorting Test (MCCST), which assessed cognitive flexibility, and the Wechsler Intelligence Scale for Children (WISC) digit span, which assessed working memory.

### Patterning Measure

**FIGURE 1:**
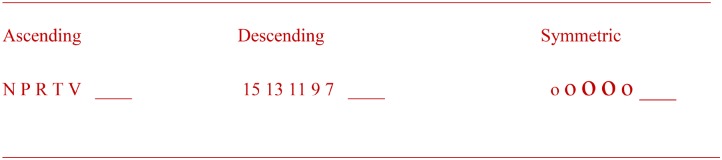
Examples of ascending, descending, and symmetric patterns. Two of each of the three kinds of patterns were made of letters, two of numbers, and two of shapes.

The patterning measure assessed children’s ability to comprehend 18 patterns well enough to fill in a missing piece for each one. There were three types of patterns involving three types of items. The 18 patterns consisted of horizontal lines of numbers, letters, and shapes that either ascended or descended in value, position in the alphabet, or size, or were symmetrical (**Figure 1**). The missing item was at the last position in the pattern. Each child was told that he or she would be shown some letters, numbers, or shapes and that one of them would be missing. Next, the children were shown the 18 patterns in a flip book, one at a time, and after each presentation the children were asked to choose the letter, number, or shape that was missing from four possible options shown below the pattern. The total number of correct responses was used in the analyses.

### Wechsler Intelligence Scale for Children (WISC) IV Digit Span

The WISC digit span was used to assess children’s working memory. The instructions for the digit span were that the researcher would read some numbers and that they must remember them and tell them back to the researcher. Children were read a series of digits and asked to say them back to the researcher. Children were provided with a practice item. First, children were asked to recall the digits provided to them exactly as recited (forward), which assesses children’s memory capacity. The forward digit span included 14 sets of three to nine numbers, with two trials for each set. For the digit span backward, children were asked whether they understood what backward meant. If they did not, an example of giving the list backward was provided to them. No other demonstration was provided. Then, children were asked to recall in backward order the digits read to them. The backward digit span included 12 sets of two to eight numbers, with two trials for each set. Testing was discontinued if a child was incorrect on both trials for sets of any given length.

The forward digit span, which assesses overall memory capacity, was used only to help the children warm up to memorizing numbers, and so was not scored. The backward digit span assesses working memory; the total number of correct trials for the backward task was used to assess working memory.

### Day–Night

The day–night inhibition test was an assessment of the child’s ability to inhibit an initial, automatic response. A flip book contained eight pictures of suns and eight of moons in a counterbalanced order. Children were asked to play a game where they were to say “night” when shown a sun and to say “day” when shown a moon. The total number of correct responses was used in the analyses. The internal reliability of the measure has been reported to be high with a Kuder–Richardson reliability of 0.93 (Chasiotis et al., 2006).

### Multiple Classification Card Sorting Test (MCCST)

This cognitive flexibility measure is part of Word Callers, an assortment of educational materials published by Heinemann Publishing. It required children to sort cards into four piles based on two dimensions simultaneously: the color and type of object on the card (e.g., sorting by yellow and brown tools and instruments; Bigler and Liben, 1992; Cartwright, 2002; Cartwright et al., 2010). The researcher introduced the task by telling children that they would be placing cards into four different piles according to the color and kind of object on the card. A set of 12 training cards was used to familiarize the child with the task. The researcher showed the children how to sort the cards by sorting the training set of cards and explaining the reasoning for each card’s placement in the training sort. Following the training sort, children were asked if they had any questions. For the test, four sets of 12 cards were presented in a random order. Children were reminded that they would need to place the cards into four different piles by color and kind of object prior to each of the four test sorts. Children were scored on accuracy of sorting (one point for correct sort), and the time (in seconds) of each sort was recorded with a stopwatch. The children were also asked to explain the reasoning for sorting the cards in the way that they did. If the sort was incorrect, the piles were corrected and the children were asked why the cards might be sorted the correct way. Children received two points for correct justification, following prior work. A flexibility composite score was calculated by adding the sorting score and justification score and dividing by the sorting time. Reliability for this measure is high with a Cronbach’s alpha of 0.86 (Cartwright et al., 2010).

### GORT

The GORT is an assessment of reading ability. Children were required to read aloud five brief stories and asked to read for comprehension. Each story is more difficult to read and comprehend than the one which precedes it. The time they took to read each story and any words read incorrectly were recorded. After each story, children were asked to answer five comprehension questions. The test was ended (ceiling was reached) after three incorrect responses to the comprehension questions on any one story. Children also received an average comprehension score: the average number of correct responses to the comprehension questions across each passage. Fluency scores for each child were obtained by calculating words correctly read per minute. Children’s reading fluency score, average comprehension score, and number of stories were used in the analyses. The reliability and validity of the GORT is high, ranging from 0.85 to 0.95 on test–retest comparisons and a median correlation coefficient of 0.63 with six other standardized measures (Kidd et al., 2014).

### Procedure

Testing was conducted during October and November. All children were tested individually during separate sessions for each measure. The order in which children received the measures was counterbalanced by classroom so that scores on the tests would not be affected by having one test which all children always took first, last, etc. All testing was completed in a quiet location within the classroom or in a quiet hallway. Each of the sessions lasted approximately 5–10 min, depending on individual performances.

## Results

Descriptive statistics were calculated for the variables of interest (**Table 1**). All dependent variables and predictor variables – except that for inhibition – were normally distributed. Correlations between patterning, reading, and EF are shown in **Table 2**. Working memory was related to both reading fluency, *r*(73) = 0.30, *p* = 0.005 and reading comprehension, *r*(72) = 0.27, *p* = 0.014, and inhibition was related to reading comprehension, *r*(72) = 0.23, *p* = 0.026. Patterning was related only to cognitive flexibility, *r*(82) = 0.31, *p* = 0.004. Cognitive flexibility was also significantly related to inhibition, *r*(82) = 0.26, *p* = 0.009, and working memory, *r*(82) = 0.34, *p* = 0.002, but the latter two EF were not correlated with each other.

**Table 1:** Descriptive statistics for variables.

	Mean	SD	Min	Max	Skew	Kurtosis
Inhibition	14.65	3.15	0.00	16.00	–3.41	12.02
Working memory	2.69	1.27	0.00	6.00	–0.31	0.17
Cognitive flexibility	0.05	0.03	0.00	0.16	0.53	0.15
Patterning	7.94	3.51	0.00	16.00	0.37	–0.21
Spatial	2.85	2.34	0.00	8.00	0.55	–0.75
Pictorial	4.21	1.88	0.00	6.00	–0.78	–0.62
Alphabetical	0.89	0.99	0.00	4.00	1.05	0.46
GORT fluency	58.99	36.89	3.74	151.28	0.72	–0.39
GORT comprehend	2.69	0.87	1.00	4.80	0.05	–0.14


**Table 2:** Correlations between variables.

	Inhibition	WM	Pattern	Fluency	Comp
Cognitive flexibility	0.26^∗^	0.34^∗∗^	0.31^∗∗^	0.09	0.15
Inhibition		0.02	–0.01	–0.15	0.23^∗^
Working memory			0.14	0.30^∗∗^	0.27^∗^
Patterning				0.08	0.09
GORT – Fluency					0.44^∗∗^


*^∗^p < 0.05, ^∗∗^*p* < 0.01. Exact *p*-values are in the text. The notation for significant *p*-values above reflect uncorrected critical values (*p* < 0.05) for these planned comparisons. If a Bonferroni correction for random comparisons was applied, the critical value for *r* would be 0.283 (*p* < 0.005).*

The value of *r* is the effect size, and those which were significant ranged between 0.23 and 0.34. Correlations between 0.10 and 0.29 are considered to be small effects: those of 0.30 and above are considered to be medium effects (Cohen, 1992).

Regression analyses were run to examine which variables predicted overall patterning performance, reading fluency and reading comprehension. For patterning, a set of sequential linear regression models was run with inhibition, working memory, and cognitive flexibility as predictor variables. Cognitive flexibility contributed significant and unique variance, *p* = 0.01, over the other two EF variables, inhibition, *p* = 0.33 and working memory, *p* = 0.33, *F*(2, 78) = 3.51, *p* = 0.02 (**Table 3**), confirming the findings from the correlations; that is, the only meaningful correlations of patterning is with cognitive flexibility. For reading fluency and reading comprehension, sequential linear regressions were conducted with inhibition, working memory, cognitive flexibility, and patterning as predictor variables. Working memory contributed the only significant variance in reading fluency, *p* = 0.02, *F*(4, 67) = 2.15, *p* = 0.08 (**Table 4**). However, both working memory, *p* = 0.08, and inhibition, *p* = 0.06, contributed unique variance for reading comprehension, *F*(4, 65) = 2.16, *p* = 0.08 (**Table 5**).

**Table 3:** Sequential linear regression predicting patterning from working memory, inhibition, and cognitive flexibility.

Predictors	*R*^2^	Adj *R*^2^	*B*	*t*
Model 3	0.12	0.09		
Working memory			0.13	1.13
Inhibition			–0.10	–0.87
Cognitive flexibility			0.29^∗^	2.56^∗^


*^∗^p < 0.05. The notation for significant *p*-values above reflect an uncorrected critical value (*p* < 0.05). If a Bonferroni correction is applied, the rejection region would be *p* = 0.01.*

**Table 4:** Factor loadings for 16 patterns within patterning measure.

Component	1	2	3	
Symmetrical number	0.43		0.26	
Symmetrical number	0.64			
Symmetrical letter	0.41	–0.21		
Symmetrical letter	0.68		–0.33	
Ascending number	0.67			
Ascending number	0.62			
Descending number	0.77			
Descending number	0.60			
Symmetrical object size			0.60	0.28
Symmetrical object size			0.34	0.30
Ascending object size			0.74	
Ascending object size			0.75	–0.23
Descending object size			0.82	
Descending object size			0.80	
Ascending letter			0.50	
Ascending letter		0.39	0.59	
Descending letter			0.40	
Descending letter		–0.20	0.63	
Eigenvalue	3.97	2.37	1.68	
% of Total variance	22.03	13.18	9.31	


*All bolded factor loadings are >0.30.*

**Table 5:** Correlations of patterning types with working memory and cognitive flexibility.

	Spatial	Pictorial	Alphabetical
Working memory	0.25^∗^	0.05	–0.20
Cognitive flexibility	0.11	0.44^∗∗^	–0.03


*^∗^p < 0.05, ^∗∗^*p* < 0.01. The notation for significant *p*-values above reflect an uncorrected critical value (*p* < 0.05). If a Bonferroni correction were applied, the critical *p*-value would be 0.01.*

### Patterning Types Factor Analysis

Subsequent analyses were conducted to examine types of patterns that emerged from the patterning measure. A principal component analysis (PCA) was conducted with a Promax oblique rotation of the 18 patterns, with restriction to three factors, due to the expectation that factors may emerge based on pattern type (e.g., object size, numbers, and letters). The Kaiser–Mayer–Olkin measure of sampling adequacy showed the sample was factorable (KMO = 0.694). The goal was to identify factors with loadings >0.30. Three factors emerged from analysis of the patterning measure explaining 45% of the variance for the entire set of patterns (**Table 6**). All patterns adequately fit into one of the three factors.

**Table 6:** Hierarchical linear regression predicting patterns from working memory, inhibition, and cognitive flexibility.

Predictors	*R*^2^	Adj *R*^2^	*B*	*t*
**Spatial patterns**
Model 1	0.09	0.08		
Working memory			0.31^∗∗^	2.87^∗∗^
Model 2	0.09	0.07		
Working memory			0.30^∗^	2.65^∗^
Cognitive flexibility			0.01	0.06
**Pictorial patterns**
Model 1	0.20	0.19		
Cognitive flexibility			0.44^∗∗^	4.43^∗∗^
Model 2	0.20	0.17		
Cognitive flexibility			0.47^∗∗^	4.41^∗∗^
Working memory			–0.08	–0.76


*^∗^p < 0.05, ^∗∗^*p* < 0.01. The notation for significant p-values above reflect an uncorrected critical value (p < 0.05). If a Bonferroni correction is applied, the critical p-value is 0.01.*

Factor 1 included number patterns that were ascending, descending, or symmetric, and letter patterns that were symmetric. The first factor, labeled spatial, explained 22.03% of the variance. Factor 2 included patterns with objects that were ascending, descending, and symmetrical in size. This second factor, labeled as pictorial, explained 13.18% of the variance. Factor 3 included only growing (ascending or descending) letter patterns. The third factor, labeled alphabetical, explained an additional 9.31% of the variance.

There were no significant predictions (*b* = 0.03, *SE* = 0.05, *t* = 0.535, *p* = 0.595; *b* = 0.05, *SE* = 0.06, *t* = 0.951, *p* = 0.345; *b* = 0.133, *SE* = 0.10, *t* = 1.270, *p* = 0.207 for the spatial, pictorial, and alphabetical factors as predictors, respectively) of reading, as measured by the average performance score on the GORT.

### Patterning Types and Executive Functions

Correlational and regression analyses were conducted to determine how the two EF that best correlated with the patterning measure were related to the different patterning types (**Table 7**). Performance on patterns spatial patterns was related to working memory, *r* = 0.25, *p* = 0.024, *R*^2^ = 0.06, whereas performance on pictorial patterns, was significantly related to cognitive flexibility, *r* = 0.44, *p* = 0.00003, *R*^2^ = 0.19. The third factor, alphabetical patterns, was not significantly related to any EF (**Table 7**).

**Table 7:** Sequential linear regression predicting reading fluency from working memory, inhibition, cognitive flexibility, and patterning.

Predictors	*R*^2^	Adj *R*^2^	*B*	*t*
Model 3	0.11	0.07		
Working memory			0.28^∗^	2.33
Inhibition			–0.17	–1.43
Cognitive flexibility			0.06	0.44
Model 4	0.11	0.06		
Working memory			0.28^∗^	2.29
Inhibition			–0.17	–1.42
Cognitive flexibility			0.05	0.37
Patterning			0.02	0.13


*^∗^p < 0.05. The notation for significant p-values above reflect an uncorrected critical value (p < 0.05). If a Bonferroni correction were applied, the critical p-value would be 0.01.*

We further examined the relations between working memory and cognitive flexibility and the two pattern types with which they were significantly correlated. We conducted hierarchical linear regressions using significantly related EF as first predictors and determined the relative contribution of all EFs using the additional EF as second predictors.

For spatial patterns, we conducted a linear regression with working memory as a predictor in Step 1 and cognitive flexibility as predictor in Step 2. Working memory significantly predicted performance, with 8% of variance explained (**Table 8**). Cognitive flexibility did not contribute additional variance, *p* = 0.96, *F*(2, 79) = 4.07, *p* = 0.02. The same amount of variance was explained when predictor entry was reversed.

**Table 8:** Sequential linear regression predicting reading comprehension from working memory, inhibition, cognitive flexibility, and patterning.

Predictors	*R*^2^	Adj *R*^2^	*B*	*t*
Model 3	0.12	0.08		
Working memory			0.23	1.80
Inhibition			0.23	1.89
Cognitive flexibility			0.04	0.33
Model 4			0.12	0.06
Working memory			0.22	1.76
Inhibition			0.23	1.90
Cognitive flexibility			0.03	0.21
Patterning			0.04	0.33


*If a Bonferroni correction is applied, the critical p-value is 0.01.*

For pictorial patterns, we conducted a sequential linear regression with cognitive flexibility in Step 1 and working memory in Step 2. Cognitive flexibility significantly predicted performance, predicting 19% of variance, *p* = 0.01 (**Table 8**). Working memory did not contribute any significant variance, *p* = 0.98, *F*(2, 79) = 10.06, *p* = 0.0001. The same amount of variance was explained when predictor entry was reversed.

## Discussion

### Reading and Patterning

The present study does not show a relation between reading, as measured by the GORT, and scores on the current patterning measure. This result does not parallel the significant correlations found for patterning and the TOWRE vocabulary measure by Schmerold et al. (2017), and the patterning and the TOWRE scores reported by Pasnak et al. (2016). This may reflect differences in these reading measures. Or, it may reflect differences in the patterning measures. Schmerold et al. (2017) used complex patterns varying in five dimensions, whereas Pasnak et al. (2016) used only alphabetic letter patterns whose items ascended by one, two, or three steps in their positions in the alphabet. Similar discrepancies in results on different reading measures – Woodcock–Johnson III scales 1, 2, and 9, the GORT, TERA, and TOWRE – have been reported for instructional interventions with complex patterning measures by Kidd et al. (2013, 2014) and Pasnak et al. (2015). It appears that the relation between reading and patterning is complex and not robust. It seems to depend on both the nature of the patterning measure used and also the reading test employed.

### EF and Patterning

A second goal of the study was to examine the relation between EF and patterning performance on a set of patterns less variable than those used by Kidd et al. (2013, 2014), Pasnak et al. (2015), and Schmerold et al. (2017). Performance on this set of patterns was correlated significantly with only cognitive flexibility. The results replicate previous findings that cognitive flexibility and patterning are significantly related for first graders (Bock et al., 2015; Schmerold et al., 2017) as well as for preschoolers (Collins and Laski, 2015; Miller et al., 2015). This suggests that cognitive flexibility is a distinctive ability that is related to understanding patterns. Patterning may involve cognitively switching one’s thinking from one possible pattern to another while completing patterning tasks.

Interestingly, neither working memory nor inhibition was related to patterning performance. This finding contradicted the findings of Borella et al. (2006) and Friedman et al. (2006) that working memory was strongly linked to patterning performance. However, Borella et al. (2006) and Friedman et al. (2006) used matrix reasoning problems in their research and had adult subjects. The difference in their results and ours may well be a function of the subjects’ ages. However, it may also be wrong to assume that the cognition used to complete linear patterns used in the current study is similar to the cognition used to complete more complex matrix reasoning problems, where there are two or three columns of stimuli involved. Although both types of problems involve detecting patterns and filling in a missing part of the pattern, there has been no research testing whether there is an overlap between these types of measures. The current research suggests that there is little overlap.

While our data did not show a relationship between patterning and working memory, Collins and Laski (2015) and Miller et al. (2015) found working memory to be significantly related to performance on alternating (repeating) patterns by preschoolers. Here, the difference in the ages of the subjects probably *is* the explanation. The current subjects were 2 to 3 years older than the preschoolers, and possessed much better working memories. It seems likely that their working memories were in excess of the task demands, and individual differences in this EF hence had little influence on accuracy in recognizing patterns of the difficulty level we employed. However, Schmerold et al. (2017) showed that with more complex and variable patterns, differences in the working memories of first graders had an effect. It is logical to conclude that the effect of working memory, if any, depends upon the interaction of the subject’s maturation and the difficulty of the task.

Lange-Kuettner and Kuettner (2015) showed that repeating the same pattern improved memory substantially, as compared with patterns that did not repeat. The patterns these researchers used were relatively unique (spatial distributions of differing shapes) and the children were older (7- and 9-year-olds). The central message as regards patterning instruction may be the same: repetitive patterns provide organization to what children perceive as otherwise chaotic collections of disparate items.

Only Collins and Laski (2015) have found an effect for inhibition on patterning. Those researchers used the Head-Toes-Knees-Shoulders (HTKS) task to measure inhibition effects on preschoolers understanding of simple repeating patterns. Miller et al. (2015) failed to find such an effect for preschoolers on repeating patterns, but used a different measure of inhibition, and Schmerold et al. (2017) found no effect for first graders on complex patterns with yet another inhibition measure It may be that the HTKS is the most sensitive measure. In any event, the current findings further indicate that for first graders, completing linear patterns of the complexity we used did not reflect differences in inhibition as measured by the Day/Night test.

### EF and Reading

Another goal was to gather more information on the role of patterning and EF in reading achievement. Previous research indicated that all three EF were related to reading comprehension (Cartwright, 2002, 2012). Additionally, it was expected that inhibition, cognitive flexibility, and patterning would be related to reading fluency (van der Sluis et al., 2007; Jacobson et al., 2011). However, the results of the present study showed that only working memory and inhibition were significantly related to reading achievement, whereas cognitive flexibility and patterning were not significantly related to either variable.

Previous research has shown that the ability to shift one’s attention and be cognitively flexible is related to multiple aspects of reading achievement, including pre-reading skills, word-reading efficiency, and reading comprehension (van der Sluis et al., 2007; Welsh et al., 2010; Cartwright, 2012). However, it has also been shown that, especially with first-grade children, a general flexibility task may not be significantly related to reading comprehension (Cartwright et al., 2010). It was previously found that reading comprehension is more related to a reading-specific flexibility task that involves switching between thinking about the sound and meaning of words rather than to a general flexibility measure that involves switching between thinking about color and shape of objects (Cartwright et al., 2010). The results of the current study further support the position that the general ability to switch one’s thinking does not strongly relate to reading achievement at this age.

Working memory was significantly related to both reading achievement variables measured: reading fluency and reading comprehension. This replicates previous demonstrations that have shown a strong relationship between working memory and multiple reading achievement variables, including reading fluency and reading comprehension (Semsa et al., 2009; Jacobson et al., 2011; Cartwright, 2012). The earlier findings show that working memory is involved at multiple levels of reading, especially reading aloud and understanding while reading. It has been posited that children with better working memory skills may have more cognitive resources to engage in the multiple processes required for reading (Semsa et al., 2009). However, more research is needed to evaluate this explanation.

Inhibition also yielded a unique prediction of reading comprehension, but not reading fluency. This finding is compatible with Cartwright’s (2012) report that inhibition relates to certain aspects of reading, including pre-reading skills, word-reading proficiency, and reading comprehension. de Beni and Palladino (2000) and Cain (2006) also found that inhibition was related to reading comprehension. This suggests that inhibition may be a necessary skill for understanding what you are reading but less important for other aspects of the process. Because their predictions of reading comprehension are unique, it appears that working memory and inhibition are explaining different aspects of reading comprehension.

### Interrelations Between EF

This project provided an opportunity to examine the interrelations between the EF variables of working memory, inhibition, and cognitive flexibility. Although these three variables have been shown to be separate, they have also been found to be correlated with one another (Miyake et al., 2000; Best and Miller, 2010). The current study showed that cognitive flexibility was significantly correlated with the other two EF; however, working memory and inhibition were not significantly related. There has been some suggestion in the research that cognitive flexibility may be comprised of these other two components and that working memory and inhibition may combine with each other to create cognitive flexibility (Garon et al., 2008; Best and Miller, 2010). The results of this study seem to support this proposition; cognitive flexibility could be a function of the combination of two variables which are relatively independent of each other.

### EF and Types of Patterns

Overall patterning performance was significantly related to cognitive flexibility; however, we found that different types of patterns related differently to EF. In particular, pictorial patterns correlated significantly with cognitive flexibility, but not with working memory. The results replicate previous findings that cognitive flexibility and patterning are significantly related (Bock et al., 2015), but also show that the relationship is due to one type of pattern. This suggests that cognitive flexibility is a distinctive EF that is needed to understand patterns in which items vary by size. Understanding these patterns may involve cognitively switching thinking from one possible pattern to another while completing pictorial patterning tasks, or it may involve switching between elements within a particular pattern. Recent research shows that when determining the next item in a sequence, preschool children may consider the abstracted labels for units of the pattern (e.g., whether it is an abab or abbabb pattern), and actually benefit from doing so (Fyfe et al., 2015). Therefore, children may be spending time switching between thinking about the abstracted labels and concrete aspects of the pictorial pattern when solving the problem. This aspect of the relation of patterning to cognitive flexibility suggests further investigation to determine whether cognitive complexity is related to other types of patterns not yet studied, and whether the same relationship applies at different ages.

Performance on spatial patterns was significantly related to working memory. Completing patterns with numbers may require continuously thinking about the numerical order of numbers and whether the numbers in a pattern are increasing or decreasing in magnitude while also thinking about symmetry and updating one’s thinking in order to complete the pattern. These results complement the findings of Lee et al. (2011), who suggested that working memory predicts patterning performance with ascending patterns of numbers. Working memory has also been linked to performance on other types of patterns, such as repeating patterns with shapes and numbers (Miller et al., 2015) and shape patterns within a matrix (Borella et al., 2006; Friedman et al., 2006). Future researchers should work to investigate the mechanisms by which working memory is related to all of these types of patterns.

The last type of pattern identified was alphabetical patterns in which the letters ascended or descended in their position in the alphabet. We found that EF were not related to performance on these patterns. This may be due to a floor effect, as these patterns were very difficult for the children. First-grade children may be too challenged by skips (missing letters) in these alphabetical sequences to use their developing EF skills to complete these types of patterns. It is interesting that the same effect was not found for patterns composed of numbers. This may be in part because children are taught in preschools and kindergartens to count by twos, fives, and tens, which involves skipping intervening numbers, but they are seldom if ever taught to skip letters as they recite the alphabet. It may also be that, as Warren et al. (2006), Clements and Sarama (2007a,b,c), and many others have suggested, patterning is most directly related to prealgebra and early mathematics.

We note that symmetrical letter patterns did not require extrapolating alphabetic sequences, because the children could simply observe the first half of the symmetrical pattern and repeat it backward.

## Conclusion

### Future Directions

Because patterning contributes to academic skills, a sensible next step may be to extend these findings to examine the relations to academic achievement of types of patterns that make different demands on EF. EF skills are related to reading comprehension (de Beni and Palladino, 2000; Cartwright, 2002; Cain, 2006), and working memory, in particular, is related to reading fluency (Locascio et al., 2010; Jacobson et al., 2011) and mathematics skills (Mazzocco and Kover, 2007; Lee et al., 2011; Jerman et al., 2012). Future research should focus on examining whether other types of patterns vary in the relation to mathematics and reading fluency and reading comprehension and whether the relationships found remain when taking into account different EF.

The finding that cognitive flexibility is an underlying factor for some patterning performance may influence future work on patterning instruction. It is known that patterning facilitates later mathematics performance and is related to some early reading skills (cf. reviews by Burgoyne et al., 2017; Pasnak, 2017). Elementary school curricula already place emphasis on detecting patterns. The present results suggest that educators may find it beneficial to also place emphasis on the switching component of completing the patterning tasks. For number-specific patterns, teachers may stress the importance of mentally manipulating the number sequence in one’s mind to complete the pattern.

Additionally, the results highlight the variables that are important for reading fluency and reading comprehension. In particular, it is important to note that both inhibition and working memory were related to reading comprehension, whereas only working memory was related to reading fluency. If methods can be devised for improving inhibition and working memory, educators may wish to focus on lessons that encourage their development when working with children who struggle with reading.

### Limitations

Although the sample was limited to children whom teachers deemed proficient in English, we had no information about bilingual status or socioeconomic status of the children. These were limitations of the study. The sample of participants was obtained from a diverse metropolitan area with children who were likely to come from families who were low in socioeconomic status or who spoke more than one language. Other researchers may wish to sample children from predominantly middle-class families to explore the generality of the results.

The children made many very low scores, particularly on the patterning measure. This was unexpected for first graders, as patterning is usually taught in preschool and kindergarten. These floor scores truncated the range of potential differences between subjects and so reduced the power of the analyses. A sample of more able children, or children assessed later in the school year, would be likely to show more variance and reduce this problem. Hence, the non-significant relations between patterning, working memory, and the GORT should be viewed with caution. This was not, however, a problem with the inhibition measure; only four of the 84 children made low scores on the Day–Night test.

Other limitations were that that the ethnicity and gender of the children were not analyzed. Ethnicity could not be determined with complete accuracy, but was in most cases clear. Gender was identifiable. Although these were not foci of this investigation or patterning research by other investigators, and differences have not emerged in such research, analyzing one or both factors could have produced additional information, and its absence is a limitation that could be avoided.

We also note that our findings are specific to children in the first grade. Our reasoning for focusing on this age was that first grade children’s patterning performance is related to reading (Kidd et al., 2014; Pasnak et al., 2015) and that first graders are beginning to comprehend a variety of pattern types. Therefore, we anticipated that we would be able to capture the EF required for understanding different types of patterns. However, our findings may not generalize to children who are beneath or beyond this age range. Additionally, we caution against generalizing these findings to other types of patterns, such as repeating or matrix patterns, or the temporal patterns explored by Lange-Kuettner et al. (2012), Lange-Kuettner and Kuettner (2015), and Hentschel et al. (2016).

This is the first study to explore the role of EF variables in performance on *different types* of patterns. The finding that cognitive flexibility and working memory have different relations to different types of patterns may influence theoretical conceptions of patterning and also future work on patterning interventions. The negative findings regarding inhibition should be regarded very cautiously. The Day–Night test was too easy and produced many ceiling scores (i.e., close to the upper limit of 16). Future research should employ a more challenging measure of inhibition. We note that it is not going to be easy to specify the relations between EF and patterning that involves different types of complex patterns, nor to relate these to different measures of prealgebra, early literacy, mathematics, and reading at different ages. Researchers interested in these variables have much to do in the years that lie ahead.

## Ethics Statement

This study was carried out in accordance with the recommendations of the Office of Sponsored Programs, Human Subjects Review Board. The protocol was approved by the Human Subjects Review Board. All subjects gave written informed consent in accordance with the Declaration of Helsinki.

## Author Contributions

All authors listed have made a substantial, direct and intellectual contribution to the work, and approved it for publication.

## Conflict of Interest Statement

The authors declare that the research was conducted in the absence of any commercial or financial relationships that could be construed as a potential conflict of interest.
